# Appropriate duration of post-surgical intravenous antibiotic therapy for pyogenic spondylodiscitis

**DOI:** 10.1186/s12879-018-3377-1

**Published:** 2018-09-17

**Authors:** Yun-Da Li, Chak-Bor Wong, Tsung-Ting Tsai, Po-Liang Lai, Chi-Chien Niu, Lih-Huei Chen, Tsai-Sheng Fu

**Affiliations:** 1 Department of Orthopaedic Surgery, Chang Gung Memorial Hospital in Keelung, Chang Gung University, 7F, No.222, Maijin Road, Keelung, 20401 Taiwan; 2 Department of Orthopaedic Surgery, Chang Gung Memorial Hospital in Linkou, Chang Gung University, Taoyuan, Taiwan

**Keywords:** Pyogenic spondylodiscitis, Recurrent risk factors, Blood culture, Spinal abscess, Parenteral antibiotic duration, Postoperative antibiotic therapy

## Abstract

**Background:**

Most guidelines recommend 6 to 12 weeks of parenteral antibiotic treatment for pyogenic spondylodiscitis. When surgical debridement is adequately performed, further intravenous antibiotic treatment duration can be reduced than that of conservative treatment alone theoretically. However, the appropriate duration of post-surgical parenteral antibiotic treatment is still unknown. This study aimed to identify the risk factors of recurrence and evaluate the appropriate duration after surgical intervention.

**Methods:**

This 3-year retrospective review included 102 consecutive patients who were diagnosed with pyogenic spondylodiscitis and underwent surgical intervention. Recurrence was defined as recurrent signs and symptoms and the need for another unplanned parenteral antibiotic treatment or operation within one year. This study included two major portions. First, independent risk factors for recurrence were identified by multivariable analysis, using the database of demographic information, pre-operative clinical signs and symptoms, underlying illness, radiographic findings, laboratory tests, intraoperative culture results, and treatment. Patients with any one of the risk factors were considered high-risk; those with no risk factors were considered low-risk. Recurrence rates after short-term (≤3 weeks) and long-term (> 3 weeks) parenteral antibiotic treatment were compared between the groups.

**Results:**

Positive blood culture and paraspinal abscesses were identified as independent risk factors of recurrence. Accordingly, 59 (57.8%) patients were classified as low-risk and 43 (42.2%) as high-risk. Among the high-risk patients, a significantly higher recurrence rate occurred with short-term than with long-term antibiotic therapy (56.2% vs. 22.2%, *p* = 0.027). For the low-risk patients, there was no significant difference between short-term and long-term antibiotic therapy (16.0% vs. 20.6%, *p* = 0.461).

**Conclusions:**

The appropriate duration of parenteral antibiotic treatment in patients with pyogenic spondylodiscitis after surgical intervention could be guided by the risk factors. The duration of postoperative intravenous antibiotic therapy could be reduced to 3 weeks for patients without positive blood culture or abscess formation.

## Background

Pyogenic spondylodiscitis is characterized by infection that primarily affects the intervertebral disc and adjacent vertebrae. The estimated incidence of spondylodiscitis, ranging from 0.2 to 10 per 100,000 inhabitants per year, has increased in recent years, which is likely associated with an aging population, higher prevalence of chronic disease, and more effective diagnostic techniques [1–3]. The pathogenesis is mostly via hematogenous seeding from distant primary infection or by contiguous spread from adjacent soft tissue [3–5]. Pyogenic spondylodiscitis is a life-threatening disease with a mortality rate of 2–20% [3, 4, 6] that is often associated with potential complications, such as paraspinal abscess, epidural abscess, meningitis, spinal instability, and neurologic deficiency [4, 5]. The relapse rates were reported to be as high as 32% in the literature [7].

The management of pyogenic spondylodiscitis is primarily a conservative treatment, comprising of long periods of parenteral antibiotics and an orthosis protection. Most guidelines recommend 6 to 12 weeks of intravenous antibiotic treatment as the standard of care in uncomplicated pyogenic spondylodiscitis [1, 5, 8–11]. Surgical interventions are reserved for patients with progressive neurologic deficits, significant vertebral destruction with instability, failure of conservative treatment, intractable pain, and for specimen collection [2–4, 6, 12]. Whereas scientific evidence is rich for conservative treatment about the optimal duration of systemic antibiotics, limited data are available regarding the appropriate duration of postoperative parenteral antibiotic treatment for patients who underwent surgical intervention. It is unclear whether there is an association between intravenous antibiotic treatment duration and treatment failure after surgical intervention. Moreover, parenteral antibiotic therapy should be limited as possible due to a prolonged intravenous antibiotic therapy increases unnecessary costs, the frequency of adverse events, and antibiotic resistance [13–15].

Theoretically, surgical debridement can eradicate the infected necrotic tissues, drain the abscess and reduce the intradiscal pressure. Adequate debridement can improve the microvascular status and allow the delivery of antibiotics more efficiently to an area of established pyogenic spondylodiscitis. We hypothesize that if surgical debridement is adequately performed, further intravenous antibiotic treatment duration can be reduced than that of conservative treatment alone. Therefore, the aims of this study were to identify the risk factors of treatment failure after surgical intervention in pyogenic spondylodiscitis and to determine the appropriate duration of postoperative parenteral antibiotic treatment after surgical intervention according to different risk of recurrence.

## Methods

### Data collection

This retrospective study was approved by the institutional review board of Chang Gung Memorial Hospital (Institutional Review Board of Chang Gung Medical Foundation Reference Number: 103-6277B). A retrospective review was conducted between January 2009 and December 2011. Adult patients (≥ 18 years of age) who were diagnosed with infectious spondylodiscitis and who underwent surgical intervention and antibiotic treatment were included. Infectious spondylodiscitis was defined using pre-operative clinical signs/symptoms, radiographic evidence, and laboratory data. Exclusion criteria included: (1) postoperative infection, (2) infection associated with prosthetic material, (3) recurrent infection, (4) tuberculous/fungal infection, (5) follow-up less than 1 year, and (6) incomplete medical records.

All patients received adequate surgical debridement and drainage according to the indications and guidelines either by the anterior or posterior spinal approach [16–19]. After surgery, organism-specific antibiotics were initiated based on the microbial report. Patients in whom the organism was not isolated received broad-spectrum antibiotics. Parenteral antibiotic treatment was usually continued until discharge, and then oral antibiotics were prescribed for at least 4 weeks according to the laboratory data in outpatient clinics.

### Definition of recurrence

Recurrence was defined as patients who had recurrent symptoms and signs such as back pain, fever, leukocytosis, and elevated C-reactive protein (CRP) and erythrocyte sedimentation rate (ESR) after a previous complete course of treatment and who needed to undergo parenteral antibiotic treatment or unplanned operation within one year.

### Study design and statistical analysis

This study included two major portions. First, we identified the independent risk factors of recurrence by multivariable analysis, using the database of demographic information, pre-operative clinical signs and symptoms, underlying illness, radiographic findings, laboratory tests, intraoperative culture results, and treatment. Categorical variables were compared using the Pearson χ2 or Fisher’s exact test. Continuous variables were compared using the Student’s t test or Mann–Whitney U test. All significant variables in the univariate analyses were subjected to multivariable logistic regression analyses to identify the independent risk factors for recurrence. Patients with any one of independent risk factors were allocated to the high-risk group of recurrence, whereas, patients with no independent risk factors were as allocated to the low-risk group of recurrence. Second, we identified the cut-off point to determine the short-term and long-term parenteral antibiotic treatment. Since some surgeons in the department believed that surgical intervention could shorten the duration of antibiotic course, a variety of complete antibiotic treatment durations were prescribed. Five different duration of postoperative parenteral antibiotic treatments (2 weeks, 3 weeks, 4 weeks, 5 weeks, 6 weeks) were assessed using Youdan’s index to obtain the best cut-off point. After statistical analysis, 3-week duration of parenteral antibiotic treatment was chosen as the cut-off point due to maximum of sensitivity and specificity. Parenteral antibiotic treatment after surgery for more than 3 weeks (> 3 weeks) was considered long-term antibiotic therapy, while treatment for less than 3 weeks (≤3 weeks) was considered short-term antibiotic therapy. Finally, we compared the recurrence rates after different durations of parenteral antibiotic treatment between the high-risk and low-risk groups. Statistical analysis was performed using SPSS 20.0 (IBM, Armonk, New York). Statistical significance was set at a *p* value of < 0.05.

## Results

**Fig. 1:**
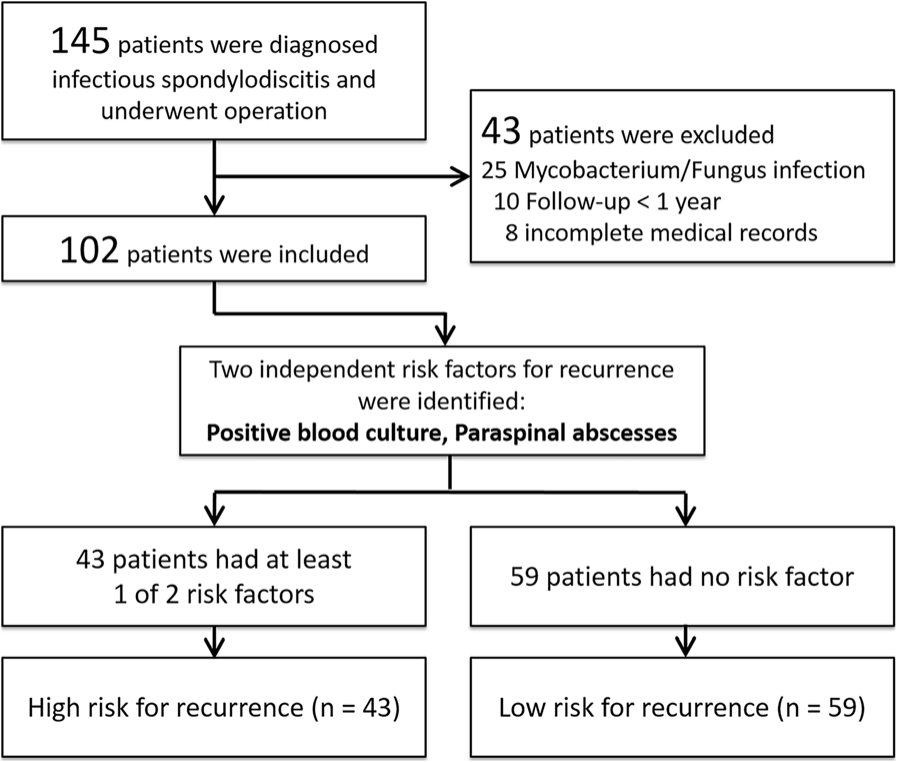
Flow chart of patients who met the inclusion/exclusion criteria for the study population

A total of 145 consecutive patients who were diagnosed with infectious spondylodiscitis and who underwent surgical treatment were identified during the study course. Of the 145 cases, 43 cases were excluded for the following reasons: mycobacterium or fungus infection (*n* = 25), less than 1-year follow-up (*n* = 10), or incomplete medical records (*n* = 8). In the end, 102 patients met the inclusion criteria and were included in this study (Fig. 1). The demographic data, underlying illness, diagnostic findings, and treatment options are shown in Table 1.

**Table 1:** Patient Baseline Characteristics

Variable	All patients (*n* = 102)
Age, year, mean (range)	61 (18–87)
Male sex	65 (63.7%)
Underlying illness	
Alcoholism	6 (5.9%)
Diabetes mellitus	33 (32.4%)
Liver cirrhosis	16 (15.7%)
End-stage renal disease	19 (18.6%)
Intravenous drug abuser	2 (2%)
Malignancy	8 (7.8%)
Clinical data	
Time to surgery, d, mean (range)	41.77 (2–180)
Back pain	95 (93.1%)
Fever	34 (33.3%)
Neurologic deficit	23 (22.5%)
Concurrent metastatic infection	32(31.4%)
Laboratory data (UNL)	
Abnormal WBC (11,500 cells/mL)	36 (35.3%)
Abnormal ESR (30 mm/h)	102 (100%)
Abnormal CRP (5 mg/L)	102 (100%)
Positive blood culture	32/85 (37.6%)
Positive tissue culture	77 (75.5%)
MRSA infection	25(24.5%)
MSSA infection	16 (15.7%)
Radiographic data	
Cervical spine infection	3 (2.9%)
Thoracic spine infection	18 (17.6%)
Lumbosacral spine infection	81 (79.4%)
More than 2 levels	20 (19.6%)
Epidural abscess	38 (37.3%)
Paraspinal abscess	22 (21.6%)
Radiographic finding	
Anterior surgery	29 (28.4%)
Posterior surgery	42 (41.2%)
A + P surgery	31 (30.4%)
Instrumentation	48 (47.1%)
Post-op IV antibiotics ≤3 weeks	41 (40.2%)
Duration of pre-op IV antibiotics, d, mean (range)	9.7 (0–60)
Recurrence	26 (25.5%)

Abbreviations: *UNL* upper normal limit, *WBC* white blood cells, *ESR* erythrocyte sedimentation rate, *CRP* C-reactive protein, *MSSA* methicillin-susceptible *Staphylococcus aureus*, *MRSA* methicillin-resistant *Staphylococcus aureus*

### Patient baseline characteristics

The median age at presentation was 61.4 years (range 18–87), and 65 patients were male (63.7%). The most common underlying illness was diabetes mellitus (32.4%), followed by end-stage renal disease (18.6%), and liver cirrhosis (15.7%). The median interval between onset of symptoms and surgery was 42 days (range 2–180). Back pain was the most common symptom (93.1%). Thirty-four patients (33.3%) had fever and 23 patients (22.5%) sustained neurologic deficits. Concurrent infection with the same pathogen occurred in 32 patients (31.4%). Urinary tract (14/32 [43.8%]) was the most common identified source, followed by pleural effusion/sputum (6/32 [18.8%]), skin/subcutaneous tissue (5/32 [15.6%]), septic joint effusion (3/32 [9.4%]), and intra-abdominal source (2/32 [6.3%]).

### Laboratory findings

Both CRP and ESR were elevated in all patients at the time of diagnosis. White blood cell (WBC) count was elevated in only 36 patients (35.3%). Blood culture was collected in 85 patients (83.3%); among them, 32 patients (38.4%) had a positive culture result for the same organism as in the surgical site culture. Successful isolation of the organism from the surgical site was possible in 77 patients (75.5%). There were still 25 patients (24.5%) with a negative culture result, and 22 (88.0%) of these 25 patients had undergone empiric antibiotic therapy prior to surgery (*p* = 0.005).

**Fig. 2:**
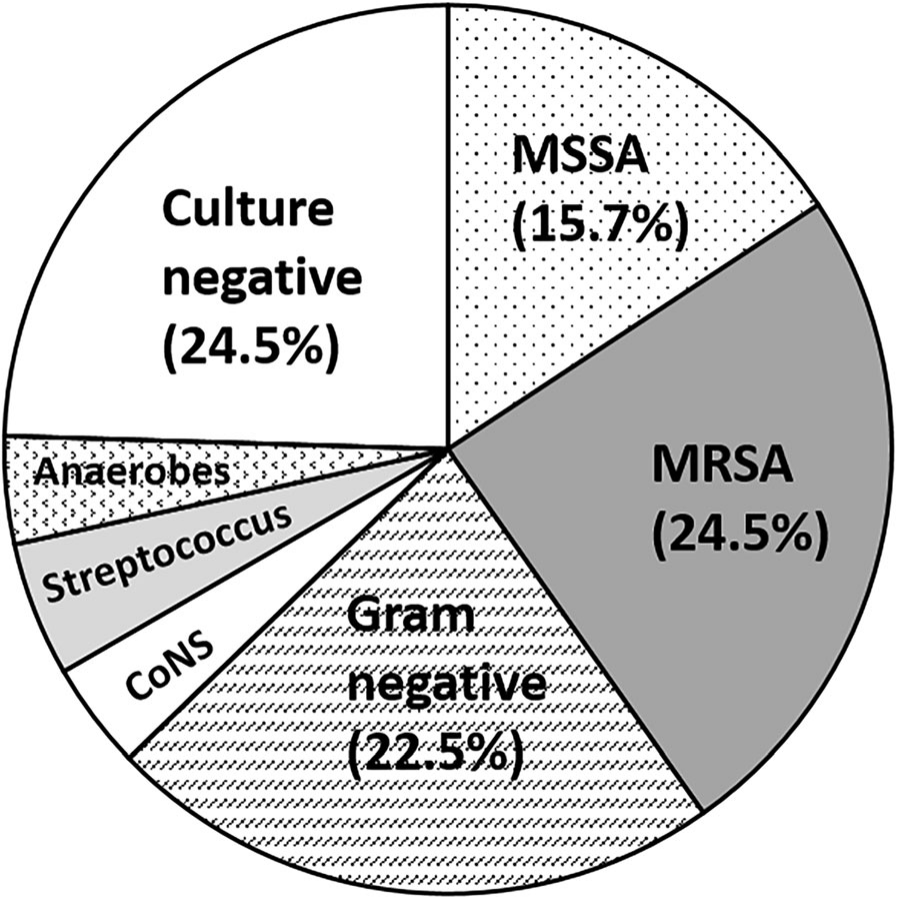
Pie chart showing the percentage of the bacterial populations isolated during surgery. Abbreviations: MSSA, methicillin-susceptible *Staphylococcus aureus*; MRSA, methicillin-resistant *Staphylococcus aureus*; CoNS, Coagulase-negative staphylococci

The most frequently isolated organisms were *Staphylococcus aureus* (41/102 [40.2%]), with 61.0% (25/41) being methicillin-resistant. Twenty-three patients (22.5%) were positive for gram-negative bacteria, 5 patients (4.9%) for *Streptococcus* species, 4 (3.9%) patients for coagulase-negative staphylococci (CoNS), and 4 patients (3.9%) for anaerobes (Table 2 and Fig. 2).

**Table 2:** Bacteria Isolated From 102 Patients During The Surgery

Organism	All patients (n = 102)
*Staphylococcus aureus*	41 (40.2%)
Methicillin-susceptible *S. aureus*	16 (15.7%)
Methicillin-resistant *S. aureus*	25 (24.5%)
Coagulase-negative staphylococci	4 (3.9%)
*Streptococcus* species	5 (4.9%)
*Viridans streptococcus*	3 (2.9%)
*Streptococcus sanguinis*	1 (1.0%)
Group B streptococcus	1 (1.0%)
Gram-negative bacteria	23 (22.5%)
*Escherichia coli*	6 (5.9%)
*Enterobacter* species	6 (5.9%)
*Klebsiella pneumoniae*	4 (3.9%)
*Pseudomonas aeruginosa*	4 (3.9%)
Other gram-negative bacteria	3 (2.9%)
Anaerobes	4 (3.9%)
Culture-negative	25 (24.5%)

### Radiographic findings

The involved area was confined to one level in 82 patients (80.4%), and more than two levels in 20 patients (19.6%). Distribution of the infection was lumbosacral in 81 patients (79.4%), thoracic in 18 patients (17.6%), and cervical in three patients (2.9%). All patients had radiographic findings of intervertebral disc space narrowing with erosion of the vertebral endplate and collapse of the vertebral bodies at the infectious level. Meanwhile, 38 patients (37.3%) had epidural abscess and 22 patients (21.6%) had paraspinal abscess accumulation.

### Treatment

Sixty-six patients (64.7%) received empiric antibiotics before the operation. The mean duration of preoperative antibiotic treatment was 9.7 days (range 0–60 days). Anterior surgery alone was performed in 29 patients (28.4%), and posterior surgery alone was performed in 42 patients (41.2%). Thirty-one patients (30.4%) underwent surgery with the combined anterior and posterior approach simultaneously or in stages. The mean period of postoperative parenteral antibiotic treatment was 24.3 days (range 5–70 days). Sixty-one patients (59.8%) received long-term parenteral antibiotics, while 41 patients (40.2%) received short-term parenteral antibiotics treatment after operation.

### Therapeutic outcomes

The median follow-up period after surgery was 27 months (range 12–67 months). Eleven patients (10.8%) suffered from the sequelae of neurologic deficits, and three patients (2.9%) died from septic shock. Among the 102 included patients, 26 (25.5%) patients developed recurrence within one year after surgery. The median time to recurrence was 110 days (range 21–330 days). Among them, four of 25 (16.0%) culture-negative cases and 22 of 77 (28.6%) culture-positive cases developed a relapse of infection. There was no statistically significant difference in the rate of recurrence between the culture-negative and culture-positive groups (*p* = 0.21).

**Fig. 3:**
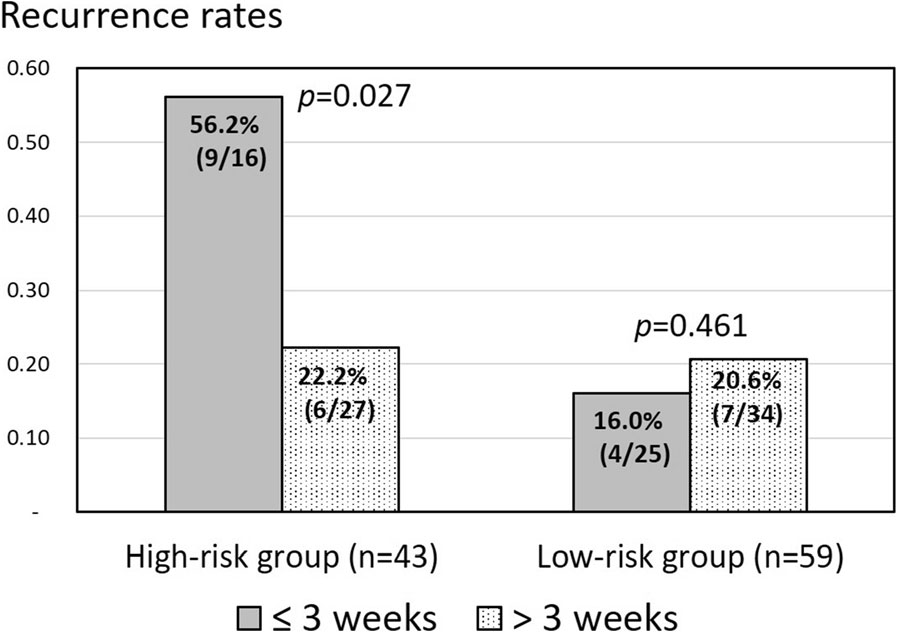
Recurrence rates in patients at low and high risk of recurrence according to the duration of parenteral antibiotic treatment after surgical intervention. There was a significant difference in recurrence according to the duration of parenteral antibiotic treatment after surgery among the high-risk patients, though there was no statistically significant difference in recurrence among the low-risk group

In the univariate analysis, diabetes mellitus (DM), positive blood culture, methicillin-resistant *S. aureus* (MRSA) infection, and paraspinal abscesses were determined to be risk factors for recurrence (Table 3). Multivariable analysis demonstrated that positive blood culture (adjusted odds ratio [aOR], 2.97; 95% confidence interval [CI], 1.13–7.91; *p* = 0.03) and paraspinal abscess (aOR, 3.36; 95% CI, 1.17–9.63; *p* = 0.02) were independent risk factors for recurrence (Table 3). Therefore, 43 patients (42.2%) with either of these two independent risk factors were classified as the high-risk group of recurrence, and 59 patients (57.8%) without any independent risk factor were classified as the low-risk group of recurrence (Fig. 1). Fifteen of the high-risk patients (15/43 [34.9%]) developed recurrence, and 11 of 59 (18.6%) low-risk patients experienced recurrence (*p* = 0.052). Among the low-risk patients, 16.0% of patients who received short-term parenteral antibiotic treatment and 20.6% of patients who received long-term parenteral antibiotic treatment developed recurrent infection within the first year after surgery. There was no statistically significant difference in the rate of recurrence between the short-term and long-term parenteral antibiotic treatment after surgery among the low-risk patients (*p* = 0.461). Among the high-risk patients, 56.2% of patients who received short-term parenteral antibiotic treatment and 22.2% of patient who received long-term parenteral antibiotic treatment developed recurrent infection within the first year after surgery. There was a significant difference in the rate of recurrence between the short-term and long-term parenteral antibiotic treatment after surgery among the high-risk patients (*p =* 0.027) (Fig. 3).

**Table 3:** Univariate and Multivariable Analyses of Risk Factors for Recurrence

Risk factors	No Recurrence (*n* = 76)	Recurrence (*n* = 26)	Univariate Analysis		Multivariable Analysis	
			OR (95% CI)	*p* value	aOR (95% CI)	*p* value
Age, y, mean	64	60	1.03 (0.99–1.07)	0.12		
Male sex	46	19	1.77 (0.66–4.72)	0.25		
Underlying illness						
Alcoholism	4	2	1.50 (0.26–8.71)	0.64		
Diabetes mellitus	20	13	2.80 (1.11–7.05)	0.03		
Liver cirrhosis	14	2	0.37 (0.08–1.75)	0.35		
End-stage renal disease	11	8	2.63 (0.92–7.50)	0.08		
Intravenous drug abuser	2	0	Not calculated			
Malignancy	5	3	1.85 (0.41–8.36)	0.42		
Clinical data						
Time to surgery, d, mean	39	51	1.01 (0.99–1.02)	0.16		
Back pain	71	24	0.99 (0.97–1.03)	0.55		
Fever	27	7	0.67 (0.25–1.79)	0.48		
Neurologic deficit	17	6	1.03 (0.95–1.07)	0.39		
Concurrent metastatic infection	21	11	1.93 (0.76–4.85)	0.22		
Laboratory data (UNL)						
Abnormal WBC (11,500 cells/mL)	25	11	1.50 (0.60–3.73)	0.48		
Abnormal ESR (30 mm/h)	76	26	Not calculated			
Abnormal CRP (5 mg/L)	76	26	Not calculated			
Positive blood culture	18	14	3.76 (1.48–9.58)	0.004	2.97 (1.13–7.91)	0.03
Positive tissue culture	55	22	2.74 (0.93–8.05)	0.21		
MRSA infection	14	11	3.25 (1.23–8.57)	0.02		
MSSA infection	15	1	0.16 (0.02–1.30)	0.06		
Radiographic finding						
Cervical spine infection	3	0	Not calculated			
Thoracic spine infection	14	4	0.81 (0.24–2.71)	> 0.99		
Lumbosacral spine infection	61	22	1.35 (0.41–4.52)	0.77		
More than 2 levels	15	5	0.97 (0.31–2.99)	> 0.99		
Epidural abscess	23	15	2.30 (0.93–5.73)	0.09		
Paraspinal abscess	11	11	4.33 (1.58–11.86)	0.003	3.36 (1.17–9.63)	0.02
Treatment data						
Anterior surgery	22	7	0.90 (0.33–2.45)	> 0.99		
Posterior surgery	30	12	1.31 (0.54–3.23)	0.65		
A + P surgery	24	7	0.80 (0.30–2.15)	0.81		
Instrumentation	39	9	0.50 (0.20–1.27)	0.18		
Duration of pre-op antibiotics, d	10.2	8.4	1.00 (0.96–1.03)	0.84		

Abbreviations: *OR* odds ratio, *CI* confidence interval, *aOR* adjusted odds ratio, *UNL* upper normal limit, *WBC* white blood cells, *ESR* erythrocyte sedimentation rate, *CRP* C-reactive protein, *MSSA* methicillin-susceptible *Staphylococcus aureus*, *MRSA* methicillin-resistant *Staphylococcus aureus*

## Discussion

**Fig. 4:**
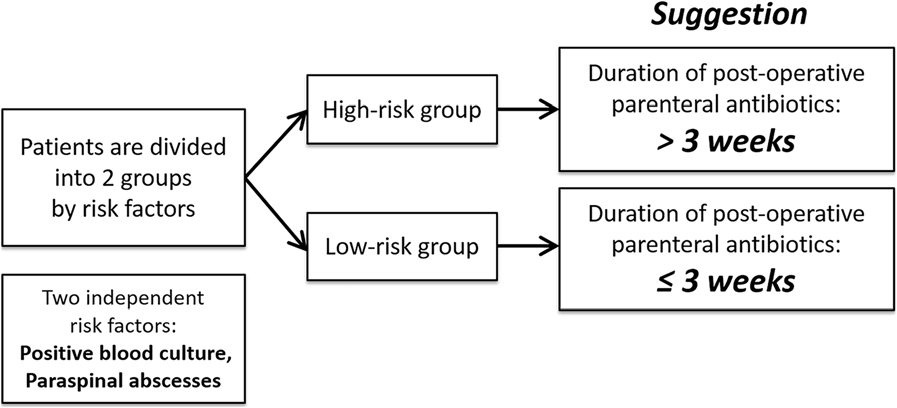
Suggestion of the optimal duration of parenteral antibiotic treatment in patients with pyogenic spondylodiscitis after surgical intervention. Patients with either positive blood culture or paraspinal abscess should be given parenteral antibiotic treatment for more than 3 weeks after surgical intervention. A short duration (≤3 weeks) of parenteral antibiotic treatment may be sufficient for the patients without risk factors

In light of the debate regarding the appropriate duration of antibiotic treatment for pyogenic spondylodiscitis after surgical intervention, this study tried to evaluate the outcomes and the risk factors for recurrence and to determine the appropriate duration of postoperative parenteral antibiotic treatment. We found that positive blood culture and paraspinal abscess formation were the two independent risk factors for treatment failure that could be used to guide the implementation of parenteral antibiotic therapy after surgical intervention. Among the low-risk patients, the recurrence rates were similar in both the long-term and short-term subgroups. However, significantly increasing recurrent rates were observed in high-risk patients who received short-term intravenous antibiotic treatment. Therefore, our findings may assist in establishing the appropriate duration of parenteral antibiotic treatment after surgical intervention during admission (Fig. 4). Patients with pyogenic spondylodiscitis with either positive blood culture or paraspinal abscess may require a long duration (> 3 weeks) of postoperative parenteral antibiotic treatment. Otherwise, a short duration (3 weeks) of postoperative parenteral antibiotic treatment may be sufficient.

Regarding the optimal duration of antibiotic treatment in the general population of patients with pyogenic spondylodiscitis, Berbari et al. [8] suggested a total duration of 6 weeks of parenteral or highly bioavailable oral antimicrobial therapy for most patients and 12 weeks of antimicrobial therapy for patients with Brucella species infection. A randomized, controlled trial by Bernard et al. [1] showed that antibiotic treatment could be shortened to a total of 6 weeks without increasing the risk of relapse, failure, and infection-related mortality. However, there is an overall lack of evidence-based data regarding the risk factors of infection relapse, which have the potential to guide decisions as to the appropriate duration of antibiotic treatment after surgical intervention.

Park et al. [20] conducted a retrospective study of 314 patients with microbiologically diagnosed pyogenic spondylodiscitis. This study reported three independent risk factors for recurrence: methicillin-resistant *Staphylococcus aureus* (MRSA) infection, undrained paravertebral/psoas abscesses, and end-stage renal disease (ESRD). According to these three independent risk factors for recurrence, all patients were classified as either low-risk or high-risk, similarly to our study. In both groups, there were significant decreasing trends for recurrence according to the total duration of antibiotic therapy. This study concluded that antibiotic therapy of long duration (≥8 weeks) should be given to patients at high risk of recurrence. For low-risk patients, a shorter duration (6–8 weeks) of pathogen-directed antibiotic therapy was deemed adequate. However, since surgical intervention was performed in only 153 (44.3%) patients in that study, it is difficult to draw conclusions regarding the appropriate duration of antibiotic treatment after surgical intervention since more than half of the cohort of patients did not undergo surgery. By comparison, our study is stronger and is more focused on the appropriate duration of antibiotic treatment after surgical intervention for pyogenic spondylodiscitis.

Recurrence rates have been reported to range from 0 to 32%, and recurrence usually occurs within 6 months to 1 year [2, 7, 13, 21, 22]. Due to variable definitions of recurrence and inclusion criteria, it is difficult to compare recurrence rates between different studies. In the current study, 26 (25.5%) cases experienced recurrence. The failure rate in our study is similar to the rates reported by Arnold et al. [23]. They indicated that treatment failure is most likely to happen within the first year and demonstrated risk factors for treatment failure including infections of the lumbar or sacral spine and prior incision and drainage. In a 10-year retrospective study, Roblot et al. [9] also proposed risk factors for relapse, which included the use of corticosteroids, rheumatoid arthritis, endocarditis, high C-reactive protein value, and a longer duration of parenteral antibiotic therapy. Due to the small number of relapses, multivariable analysis was not performed in their study. McHenry et al. reported that recurrent bacteremia, paravertebral abscesses, and chronically draining sinuses were the independent risk factors associated with relapse [21]. Notably, in our study, diabetes mellitus (DM), end-stage renal disease (ESRD), methicillin-resistant *Staphylococcus aureus* (MRSA) infection, and epidural abscess showed a trend for higher risk of recurrence but didn’t achieve statistical significance. This result might be partially attributable to the relatively low number of participants in our study. There should still be special consideration for patients with these factors in clinical practice.

Surgical debridement provides eradication of infectious tissues and adequate tissues for culture to determine the type of bacteria, which informs the choice of appropriate antibiotics for optimal infection control. In theory, this could shorten the duration of the antibiotic course and reduce immobilization-related complications [24, 25]. Although a wide spectrum of organisms has been related with pyogenic spondylodiscitis, *S. aureus* is the predominant organism, accounting for around half of the cases (range 20–84%) in the literature [2, 11, 21, 22, 26, 27]. Enterobacteriaceae, such as *E. coli*, *Proteus*, *Klebsiella*, and *Enterobacter* spp., have accounted for 7–33% of pyogenic spondylodiscitis cases [2, 21, 22, 26, 27]. In addition, streptococci, enterococci, and coagulase-negative staphylococci (CoNS) are also well-described organisms that account for 5–20% [2, 11, 21, 26, 27]. Anaerobic bacteria rarely cause spondylodiscitis and are associated with less than 4% of cases [2, 11, 21, 26]. The findings of our study were in accordance with these rates of causative organisms.

Since pyogenic spondylodiscitis can be treated with conservative treatment [28–30], patients often receive empiric antibiotic treatment before a definite culture result has been obtained. In the current study, 66 patients (64.7%) received parenteral antibiotic treatment prior to surgery. However, culture yield may be decreased by antibiotic use before biopsy or surgery [2]. Among the 25 culture-negative cases in the current study, 22 (88%) had received antibiotic therapy before surgery (*p* = 0.005), which showed that a negative culture result is significantly associated with preoperative antibiotic treatment.

Our study had some limitations. Due to the retrospective design of the study, some important clinical characteristics may not have been recorded and some patients were lost to follow-up, which may have introduced unrecognized bias. There was also a selection bias of patients by their treating physicians, since the duration of intravenous antibiotic treatment was decided according to clinical and biological response to treatment in this retrospective study. In addition, this study included patients with both microbiologically proven and culture-negative cases. Hence, the included cases were of clinically defined pyogenic spondylodiscitis, and not all were microbiologically diagnosed, which may have also introduced unrecognized bias. Another potential limitation may be that the use of oral antibiotics was not included in our study, because we wanted to emphasize hospitalized treatment courses; additionally, patient compliance with oral antibiotics in the outpatient clinic can be difficult to control.

## Conclusions

Positive blood culture and paraspinal abscess formation were found to be the independent risk factors for recurrent infection in pyogenic spondylodiscitis. Our results suggest that the appropriate duration of parenteral antibiotic treatment in patients with pyogenic spondylodiscitis after surgical intervention should be guided by the recurrence risk. Parenteral antibiotic therapy should be given for more than 3 weeks to patients with either of the two risk factors for recurrence. For low-risk patients, a 3-week short-term parenteral antibiotic treatment may be sufficient.

## Abbreviations

CoNS: coagulase-negative staphylococci
CRP: C-reactive protein
DM: diabetes mellitus
ESR: erythrocyte sedimentation rate
ESRD: end-stage renal disease
MRSA: methicillin-resistant *S. aureus*
WBC: White blood cell
